# Pre-Gelatinisation of Rice Flour and Its Effect on the Properties of Gluten Free Rice Bread and Its Batter

**DOI:** 10.3390/foods10112648

**Published:** 2021-11-01

**Authors:** Xiang-Li Ding, Lan-Jing Wang, Ting-Ting Li, Fei Wang, Zhen-Yang Quan, Meng Zhou, Zhong-Yang Huo, Jian-Ya Qian

**Affiliations:** 1 Key Laboratory of Chinese Cuisine Intangible Cultural Heritage Technology Inheritance, Ministry of Culture and Tourism, School of Food Science and Engineering and School of Tourism and Culinary Science, Yangzhou University, 196 West Huayang Ave., Yangzhou 225009, China; dingxl@yzu.edu.cn (X.-L.D.); 182405119@yzu.edu.cn (L.-J.W.); MZ120191369@yzu.edu.cn (T.-T.L.); 182405117@yzu.edu.cn (F.W.); MX120211226@yzu.edu.cn (Z.-Y.Q.); xuyanlp@yzu.edu.cn (M.Z.); 2 Co-Innovation Center for Modern Production Technology of Grain Crops, Jiangsu Key Laboratory of Crop Genetics and Physiology, Research Institute of Engineering Technology for Crop Industry, Yangzhou University, 196 West Huayang Ave., Yangzhou 225009, China; huozy69@163.com

**Keywords:** pre-gelatinisation, rice flour, gluten-free, rice bread

## Abstract

In order to improve the quality of the gluten free rice bread (GFRB), pre-gelatinised rice flour (PGRF) was made and used to partially replace natural rice flour in the production of GFRB. The pre-gelatinisation parameters were optimised and the effects of PGRF on the quality of the GFRB and its batter were studied. The results showed that optimal PGRF was obtained when 50% total water was mixed with 1.0% rice flour and the mixture heated at 80 °C for 2 min. Supplementation with PGRF significantly improved the properties of GFRB by affecting its baking properties, textural properties, colour, and crumb grain features. Effects of PGRF on GFRB were mainly caused by the more closely packed gel structure of rice starch in the bread batter, the higher onset temperature during gelatinisation and the complex effect of PGRF on water-binding capacity in bread batter during the baking process. As the pre-gelatinisation parameters of flours and their effect on gluten-free baked products varied with grain variety, processing properties should be studied before using them, and emphasis should be placed on new techniques such as flour pre-gelatinisation to obtain gluten-free foods with improved quality.

## 1. Introduction

In 2005, Gallagher et al. [1] predicted that the global incidence of coeliac disease (CD) would increase by a factor of 10 over the following years. Research by Kang et al. [2] also demonstrated this increase in the gluten-free (GF) product market and attributed it to the advent of serological testing and people’s increased awareness of CD. The global GF products market size is estimated to account for USD 5.6 billion in 2020 and is projected to reach USD 8.3 billion by 2025 [3]. The increasing prevalence of CD, combined with increases in the prevalence of other allergic reactions and intolerances to gluten, is expected to further increase the need for high-quality GF products in the future [4,5].

The characteristics of rice flour, including its neutral flavour, easy digestibility, and hypoallergenic proteins, make it a suitable raw material for producing gluten-free rice bread (GFRB). Extensive research has been performed on GFRB, with most studies focusing on the influence of raw materials on GFRB quality, and approaches to improve the properties of GFRB. Research on raw materials from rice has shown that the variety [6], flour particle size [7], physicochemical properties [8], and starch diversity [9] are all related to GFRB quality. The approaches applied to improve the properties of GF bread can be categorised as ingredient addition and technological. Extensive research has been performed on ingredient addition strategies, including the use of various hydrocolloids or thickening agents [10], non-gluten proteins [11], fats and low-molecular-weight carbohydrates [12,13], hydrocolloid-emulsifier-protein systems and enzymes [14], with the aim of improving the textural properties and consumer acceptance. In comparison, much less research attention has been given to technological approaches. Recently, the consumer demand for clean labels has redirected researchers to alternative technological approaches that allow the production of high-quality GF bread without the need for added ingredients. In this context, non-conventional baking methods using high hydrostatic pressure and sourdough fermentation have been developed and are increasingly used in the baking industry [14]. Non-conventional baking techniques include microwave, infrared, and jet-impingement heating or a combination of these techniques (hybrid heating). Among these techniques, hybrid heating techniques using infrared-microwave baking [15] and Ohmic heating [16] have been investigated in GF bread baking. Although these techniques have shown promising results, drawbacks, such as higher crumb firmness and moisture loss, lower starch granule disintegration, decreased gelatinisation or digestibility, and increased flavour loss still impede their implementation at an industrial scale [15]. The application of high hydrostatic pressure for the production of GF bread may increase its viscoelastic properties and thus improve bread volume and texture. Cappa et al. [17] found that optimal sourdough fermentation conditions improve the sensory, nutritional, and textural qualities of GF bread [17]. These improvements, which are simultaneously cost-effective and environmentally friendly, make sourdough fermentation the most promising technological approach for the production of GF bread. However, the choice of appropriate starter cultures for various raw materials is crucial for consistent bread quality, which makes the industrial application of this technique complex [14]. The National Food Centre of Ireland has performed preliminary research studies on a number of commercially available GF breads [1]. These breads were found to be of lower quality than their gluten-containing counterparts. Research groups have, therefore, embraced the use of novel ingredients to improve the quality and sensory attributes of GF bread formulations and to study their effects on dough and batter rheology and baking characteristics [1]. Thus, the demand for novel approaches to improve the quality of GF bread remains pressing. 

Pre-gelatinised starches, obtained by heating in the presence of water, are widely used for their technological properties, such as solubility in hot or cold water, high viscosity, and smooth texture [18]. They can be used in food processing when thickening is required to improve texture, such as in cereal porridge, soups, and creams. Rice porridge was used to cook rice bread in the 18th century. Tsai et al. [19] confirmed that rice porridge affects the viscoelastic properties of the dough and the texture of the bread during storage. Later, the effects of pre-gelatinised flour or starch, and of another form known as water roux starter, were investigated in the production of breads and cakes [20,21,22]. The results of these studies showed that these ingredients improve the technological and the visual and sensory properties of the bread and cakes and delay staling. 

Recently, the use of pre-gelatinised flour, such as wheat, maize, and corn flour, has been investigated in GF bread production to satisfy the consumers’ preference for soft and glutinous characteristics [14,23,24]. However, information regarding the effect of flour pre-gelatinisation on the quality of GF bread remains scarce. Moreover, due to the variations in raw materials and its processing methods, the reported pre-gelatinisation parameters and the amount of pre-gelatinised flour included in the recipe have varied. Thus, in this study, pre-gelatinisation parameters were optimised using a commercial rice flour. Then, based on the optimal pre-gelatinisation parameters, the effect of pre-gelatinised rice flour (PGRF) on the properties of GFRB and its batter were investigated.

## 2. Materials and Methods

### 2.1. Materials

Wet-milled rice flour (Erawan Brand, Cho Heng Rice Vermicelli Factory Co., Ltd., Nakhon Pathom, Thailand) was produced by Cho Heng Rice Vermicelli Factory Co., Ltd. Hydroxy propyl methyl cellulose (ZW-HPMC-2910E15, Mizuda Bioscience Co., Ltd., Huzhou, China) was provided by Mizuda Bioscience Co., Ltd. Sugar (Shanghai Sugar Tobacco and Alcohol Co. Ltd., Shanghai, China), salt (Jiangsu Ruifeng Salt Industry Co., Ltd., Xuzhou, China), dry yeast (saf-instant baker’s yeast, Guangzhou Danbaoli Yeast Co., Ltd., Laibin, China) and refined soy oil (Yihaikerry Food Marketing Co., Ltd., Yingkou, China) were purchased from the local market. 

### 2.2. Batter Preparation and GFRB Making

The dough was performed using the modified formula reported by Marco et al. [25] which contained soy oil (6 g), sugar (5 g), salt (2 g), instant yeast (2 g), hydroxy propyl methyl cellulose (4 g), and water (110 g) at the base of 100 g rice flour. A straight-dough process was used for control samples. In samples with PGRF, part of the rice flour was heated in water in a constant temperature heating magnetic stirrer (DF-101S, Shanghai Lichen Bangxi Instrument Technology Co., Ltd., Shanghai, China) with constant stirring. After pre-gelatinisation, the rice flour and water mixture was left to cool down to room temperature then mixed with the rest of the rice flour and the other ingredients in the formula. Part of GFRB batter was freeze-dried for further analysis, and 100 g batter was put into pans (16.6 cm × 6.5 cm × 3 cm) for fermentation. Fermentation was conducted for two steps. First, it was fermented for 38 min at 35 °C and 85% relative humidity. Then, it was stirred with a fork for 1 min to break up the large bubbles in the batter and left to continue to ferment until its height was flat with the edge of the pan. Water was sprayed on the surface, and then it was baked in an electric oven (SINMAG SM-523, Sinmag Equipment (China) Co. Ltd., Wuxi, China) at 210/210 °C. In preliminary work, a sticky and wet crumb was obtained in samples with PGRF when the same baking time was used as for the control samples. This texture made it difficult to slice the bread, and the surface of the crumbs was too rough to analyse. Thus, the baking time for control and PGRF samples was set to be 25 min and 27 min, respectively, to obtain bread samples which could be sliced for further analysis. After baking, the GFRB was demoulded, cooled for 50 min at room temperature, and packed with polyethylene bags for bread properties determination. Slices were cut into 1 cm thick from the centre of bread sample using a mechanical slicer (SM302N, Sinmag Equipment (China) Co. Ltd., Wuxi, China) for crumb properties determination.

### 2.3. Pre-Gelatinisation Parameters Determination

Pre-gelatinisation parameters including the pre-gelatinisation temperature, substitution ratio of PGRF (rice flour base), ratio of water used for pre-gelatinisation (total water used in batter base), and pre-gelatinisation time was determined using the specific volume as index. Pre-gelatinisation parameters and levels of rice flour are listed in Table 1. After optimisation, GFRB and its batter was prepared according to the optimal parameters for properties determination.

### 2.4. Properties Determination of Gluten Free Rice Bread

#### 2.4.1. Baking Properties

Baking properties including the specific volume, baking loss, and moisture content of breadcrumb were determined. 

The weight and loaf volume of GFRB samples was determined by rapeseed displacement after being cooled for 50 min at room temperature. Then, the specific volume (mL/g) of baked bread was calculated as quotient of loaf volume (mL) and weight (g) of each bread sample.

Baking loss was the change of weight before and after baking. It was calculated with baking loss (%) = (weight of batter before baking-weight of bread after baking)/weight of batter before baking × 100.

The moisture content of breadcrumbs was determined using an infrared moisture tester (WY•105W, Shanghai Wanyuan Electronic Technology Co., LTD., Shanghai, China).

#### 2.4.2. Texture Properties of Breadcrumbs

The texture properties determination of the breadcrumbs was conducted after 24 h (packed with polyethylene bags, 25 °C) of baking using a TA-XT2 texture analyser (Stable Micro System Co. Ltd., Surrey, UK) equipped with a P/36R Aluminium Platen probe of 2.5 cm diameter. Bread slices were compressed up to 30% at a speed of 2 mm/s. One bread slice was used at a time in textural analysis and texture properties were determined at least eight times for each bread sample. 

#### 2.4.3. Crumb Colour Determination

The crumb colour of GFRB was determined by an automatic whiteness metre (ACDI-2000, Beijing Chentech Instrument Technology Co., Ltd., Beijing, China). The automatic whiteness metre was calibrated with a black barrel and a white board, respectively. Then breadcrumbs were cut with an annular mould and then inserted into the automatic whiteness metre and placed on the light source. L*, a*, and b* values were recorded. At least ten crumb slices were measured in each group.

#### 2.4.4. Crumb Grain Features Observation of Bread

Bread slices were scanned using a scanner (LaserJet Pro MFP M227fdw, Hewlett-Packard, Tokyo, Japan) at a resolution of 600 dpi. A field of view 165 × 165 was cropped from the crumb grain feature image using Adobe Photoshop CS5. The cropped images were converted from RGB mode into grey scale, converted into binary image, and then analysed with MATLAB 2007b software program (Program S1) using the slightly modified image analysis method described by Ding [26]. Average area, number, ratio, and total area of crumb grain pores were quantified and recorded.

### 2.5. Determination of Properties of GFRB Batter

#### 2.5.1. Microstructure Measurement of GFRB Batter

GFRB batter was freeze-dried, fractured, and sputter coated with gold-palladium alloy. Then, the microstructure of gluten free rice bread batter was observed with SEM (GeminiSEM 300, Carl Zeiss, Oberkochen, Germany) under vacuum and at an accelerating voltage of 5 kV. Representative micrographs were taken for each sample at magnification of ×3000. 

#### 2.5.2. Gelation Properties Measurement of GFRB Batter

The GFRB batter was freeze-dried, fractured, pre-hydrated with water at ratio of 1:2 (3 mg:6 mg), and equilibrated at 4 °C for 24 h in sealed aluminium pan and gelation properties of gluten free rice bread batter was measured with DSC (DSC200F3, Netzsch Co., Ltd., Bavaria, Germany). Samples were scanned from 25 °C to 100 °C at a heating rate of 5 °C/min. The enthalpy of the gelatinization (∆Hg, J/g) and onset, peak, and final temperature of each sample was calculated from its gelatinisation curve.

#### 2.5.3. Thermogravimetric Analysis/Simultaneous Differential Thermal Analysis (TGA/SDTA) of GFRB Batter

The GFRB batter was freeze-dried, fractured, and pre-hydrated with water at ratio of 1:1.1 and equilibrated at 4 °C for 24 h. TGA was carried out with a TGA/SDTA (Pyris 1 TGA, PerkinElmer, Waltham, MA, USA) in a nitrogen atmosphere. Samples (3 mg) were heated from 25 °C to 300 °C at a heating rate of 10 °C/min with N_2_ at 20.0 mL/min. 

### 2.6. Statistical Analysis

The data shown are expressed as mean ± SD. The Origin software was used in the data treating and diagram making. Statistical analyses were carried out using SPSS for Windows, version 16.0 (SPSS Inc., Chicago, IL, USA, 1999). All statistical analyses were done by one-way analysis of variance (ANOVA) with Duncan post hoc test. The significance level of *p* < 0.05 was used.

## 3. Results and Discussion

### 3.1. Effect of Pre-Gelatinisation Parameters on the Specific Volume of GFRB

**Figure 1:**
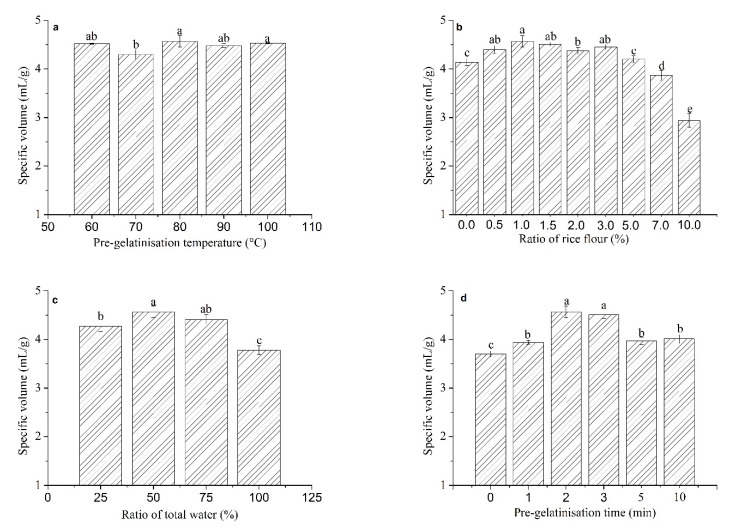
This effect of pre-gelatinisation parameters on the specific volume of gluten-free rice bread. (**a**) Pre-gelatinisation temperature; (**b**) ratio of rice flour; (**c**) ratio of used water to total water; (**d**) pre-gelatinisation time. Different letters (^a^, ^b^, ^c^, ^d^ and ^e^) on top of bars in the same graph indicate significant differences between mean values (*p* < 0.05).

About 10% of the worldwide population who suffers from celiac disease, wheat allergy, and non-celiac wheat sensitivity follow a GF diet [27], and GF bread is the most required product by them. However, commercially available GF breads were found to be of low quality. The specific volume is one of the most important indicators of bread quality [27], strongly influencing consumer choice. Thus, it was used as an indicator of GFRB quality during the optimisation of pre-gelatinisation parameters of rice flour to evaluate the effect of pre-gelatinisation parameters on bread quality. As shown in Figure 1a, an increase in pre-gelatinisation temperature from 60 °C to 80 °C significantly increased the specific volume of GFRB (*p* < 0.05), whereas no significant change was observed when the temperature was increased from 80 °C to 100 °C (*p* > 0.05). Thus, 80 °C was chosen as the optimal pre-gelatinisation temperature for rice flour. The use of PGRF had a clear influence on the specific volume of GFRB (Figure 1b). No significant difference in specific volume was observed when the amount of PGRF was increased from 0.5% to 1.5% (*p* > 0.05). The highest specific volume was obtained with 1.0% PGRF. Further increases in the amount of PGRF from 3.0% to 10.0% resulted in a significant decrease in the specific volume (*p* < 0.05). The ratio of used water to total water in the batter and the pre-gelatinisation time also had a significant effect on the specific volume of GFRB (*p* < 0.05). The largest specific volume was obtained when 50% total batter water was used for pre-gelatinisation (Figure 1c) and incubated for 2 min (Figure 1d). Various flour:water ratios have previously been used for flour pre-gelatinisation. When using the traditional water roux starter, wheat flours are mixed with water at a ratio of 1:5 (w/w) and heated at approximately 65 °C for a few minutes or mixed with half of the dough water that has been boiled [22]. Both of these techniques aim to heat the mixture until the temperature at the centre reaches 65 °C. These techniques have also been used in the production of GFRB [8]. In maize bread production, 3 g of maize flour is mixed with 8 mL of distilled water, and the pre-gelatinised maize flour is used in the dough at 30% [23]. Meanwhile, for corn bread, 120 g of corn flour is mixed with 200 mL of water for 5 min and the mixture is placed in a hot bath at 55 °C. The mixture is then extracted and allowed to stand for 10 min until the starch is deposited in the jar [18]. The levels of hydrothermally treated pre-gelatinised slurries are fixed at 14.75% and 13.8% for rice and corn breads, respectively [21]. The differences in pre-gelatinisation methods used for various flours may be due to the differences in physicochemical characteristics, pasting properties, and morphological properties of cereal grains.

The size of the starch granules of flour is a critical parameter affecting the gelatinisation of starch. In general, the gelatinisation temperature of small starch granules is higher than that of large granules due to their compact internal structure. The size of wheat and rice starch granules has been reported to range from 6.9 to 34.8 μm and 1.5 to 8.9 μm, respectively. Wheat starch is completely gelatinised at 65 °C [28], whereas the pasting temperature of rice starch is reported to be 78.75 °C [29]. The milling methods, protein content, amylose content, and shape of the starch granules of flour also affect the pasting temperature [30,31]. Alfauomy et al. [29] compared the pasting properties of starches extracted from rice and wheat and showed that the peak, breakdown, final, and setback viscosities of rice starch are higher than those of wheat starch. This may explain why only 1.0% PGRF gave optimal GFRB results, whereas pre-gelatinised wheat flour can be used at 5–10% in wheat bread making. The water-absorption capacity and swelling power of rice starch are much higher than those of wheat starch [29], and thus, more water is needed during pre-gelatinisation of the former. Sufficient time should be given for flour pre-gelatinisation to improve the physicochemical and rheological properties of the flour. However, a longer pre-gelatinisation time may result in undesirable changes in the water-holding capacity and pasting properties of the flour, which result in a decrease in the specific volume of the GFRB [18]. No significant difference was observed in the specific volume of GFRB when pre-gelatinisation times were 2 and 3 min (*p* > 0.05), and the highest specific volume was obtained at 2 min. Thus, according to the above results, the optimal PGRF was obtained when 50% batter water was mixed with 1.0% rice flour, and the mixture was heated at 80 °C for 2 min.

### 3.2. Baking Properties

The effects of pre-gelatinisation on the baking properties of GFRB are shown in Table 2. The addition of PGRF significantly increased the loaf volume and specific volume of GFRB (*p* < 0.05). A similarly high specific volume was also obtained when rice porridge was used in the production of rice flour bread [19] and when pre-gelatinised maize [23] and corn flour [24] were added to GF bread. Moreover, Tsai et al. [19] reported that the addition of gelatinised rice, in the form of gelatinised rice flour or rice porridge, to dough materials increases loaf volume and crumb softness. The inclusion of water roux starter significantly increases the loaf specific volume in an amount-dependent manner (*r* = 0.999 and *p* < 0.001) [22]. Flour pre-gelatinisation can improve the functional and handling properties of dough through increased cohesion, elasticity, and viscosity, and consequently increased gas retention [32]. It is assumed that a higher specific volume results from the increased pore area generated by the water roux starter, rice porridge, or pre-gelatinised starch [6], and that gas retention in GF batters is mainly controlled by starch gelatinisation [14]. Pre-gelatinised starches may help sustain gas pressure on the pore walls when expansion occurs during fermentation and during the early stage of baking, resulting in bread with good expansion characteristics [33].

During the production of GF pan bread, the incorporation of pre-gelatinised corn flour resulted in a higher moisture content compared to the moisture content of bread made with natural corn flour [24]. Moisture content has been shown to increase from 37.37% (control) to 39.11% and baking loss from 5.48% (control) to 3.75% when using a 6.0% water roux starter. Moisture content of the bread has also been shown to increase as more water roux starter is added (*r* = 0.999 and *p* < 0.001) [22]. Pre-gelatinised starch acts as a hydrocolloid in bread dough, as the disrupted crystal structure of the starch granular increases the connections between water molecules and starch chains. Thus, pre-gelatinised starch holds more water than regular starch due to the hydroxyl groups of the hydrocolloidal structure, which enable greater interaction with water molecules. Moreover, the increase in dough viscosity caused by the intrinsic viscosity of pre-gelatinised starch also affects moisture migration from the crumb to the crust during bread baking, resulting in less water loss and consequently, a higher moisture content. However, no obvious effect on moisture content was reported when rice porridge was used in bread making [19]. Significantly less baking loss was observed for the pre-gelatinised samples (*p* < 0.05), but no significant effect was observed on the moisture content of breadcrumbs (*p* > 0.05). Different trends in the loss of batter during baking and in the moisture content of breadcrumbs may have been caused by the inhomogeneous water distribution of the bread crust and crumbs. Moreover, a preliminary study showed that sticky, wet crumbs were obtained in samples with PGRF when using the same baking time used for the control samples (data not shown). This texture made it difficult to slice the bread, and the surface of the crumbs was too rough to analyse. Thus, prolonged baking time was applied for samples with PGRF, which caused further baking loss and led to a decreased moisture content in breadcrumbs when compared to data from the literature.

### 3.3. Texture Properties

Texture is a major criterion in assessing the eating quality of bread because of its close association with consumers’ perception of freshness. The effect of the PGRF on the texture properties of GFRB is shown in Table 3. The hardness of GFRB was significantly decreased by the inclusion of PGRF (*p* < 0.05). A decrease in hardness has been commonly reported in bread made with pre-gelatinised starch [24]. This positive effect has been ascribed to an increase in moisture retention in the final product and in certain technological properties, such as the volume and porosity of the breadcrumbs, as a high correlation has also been found between loaf size and bread softness [19]. Negative relationships have been reported between bread hardness and bread volume (*r* = −0.991, *p* = 0.001) and specific loaf volume (*r* = −0.993, *p* = 0.001) [21]. That is, samples with a higher moisture content, volume, and porosity are expected to have lower firmness [24]. For the GFRB produced in this study, the increased loaf volume and porosity was presumed to be mainly due to the decreased hardness of the pre-gelatinised samples. Adhesiveness is defined as the force required to remove breadcrumbs that adhere to the palate during mastication. Crumb adhesiveness is significantly higher in bread containing rice porridge than in bread made from wheat flour [19]. However, we observed no significant difference in adhesiveness with or without the addition of PGRF. The relatively small amount (1.0% of flour basis) of PGRF added and the lack of a significant change in the moisture content of the breadcrumbs caused by prolonged baking time may explain these results. No significant difference in springiness was reported when a water roux starter was used at various levels [22]. However, in sponge cakes, springiness is decreased in samples prepared with gelatinised starches compared to those prepared with native starches [34]. A significant decrease in springiness was also observed in this study when PGRF was used (*p* < 0.05). Osella et al. [35] declared that the springiness of GF bread is greatly affected by moisture content, moisture redistribution, and the retrogradation of starch [35]. As no significant difference in moisture content was observed between samples with or without the addition of PGRF, the decreased springiness of the bread may have been caused by the characteristics of the pore structure. Chewiness is defined by the relationship: hardness × cohesiveness × elasticity. Thus, the decrease in chewiness of GFRB with the addition of PGRF was in accordance with the decrease in hardness discussed above, even though a chewy mouth feel has been reported when rice porridge is added to bread [19].

### 3.4. Crumb Colour

Pre-gelatinised starches have various effects on the colour of baked foods. Bajaj et al. [34] investigated the effect of gelatinised starches from various sources (corn starch, waxy corn starch, potato starch, and rice starch) on sponge cake. They found that the incorporation of gelatinised starches significantly affected the colour of both the crust and crumbs. Gelatinised starch was found to decrease the L* values and increase the a* values of the crust and increase the a* values of the crumbs. Jalali et al. [24] found that pre-gelatinised corn flour significantly decreases the L* and b* values and increases the a* values of bread crust. It also significantly decreases the L* values and increases the a* and b* values of breadcrumbs [24]. However, no significantly difference in these values was observed when water roux starter was used in rice pan bread [22]. Previous studies have shown significantly lower L* and b* values in the crust of rice porridge bread than in the crust of rice flour bread, although both types of bread loaves have a golden-brown crust. The same trends in crumb colour were observed when PGRF was used to make GFRB in this study (Table 4). Differences in colour can be caused by two reasons. First, PGRF affects the porosity of the breadcrumbs, and consequently influences light reflection by the breadcrumbs and breadcrumb brightness. For example, Purlis et al. [36] reported that regular smooth surfaces result in a higher L* values compared to folded surfaces. PGRF may also affect the reducing sugar content and thus the colour formation via the Maillard reaction [19]. The addition of PGRF may also affect the temperature of breadcrumbs, which is a critical factor for the Maillard reaction, by maintaining moisture during baking [23].

### 3.5. Crumb Grain Features

**Figure 2:**
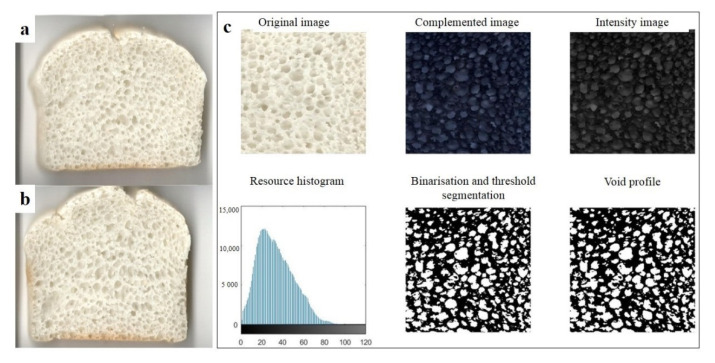
Effect of pre-gelatinisation on the breadcrumb grain feature of gluten-free rice bread. (**a**) Picture of control breadcrumb; (**b**) picture of breadcrumb added PGRF; (**c**) convention, identification, and analysis of the image of pore distribution in breadcrumb.

According to Armero et al. [37], the porosity of bread indicates the number of pores in the crumbs and it affects consumer acceptance. Porosity is determined by the number of gas cells and their size and distribution. Flour pre-gelatinisation can affect gas retention in maize bread by improving the cohesion, elasticity, and viscosity of the dough [38]. Fewer cells with an irregular shape and size have been observed in breadcrumbs containing pre-gelatinised maize flour. Furthermore, pore cell walls are very thick and composed of clumps of starch granules in these breadcrumbs [23]. Magnetic resonance imaging and scanning electron microscopy were used by Naito et al. [33] to analyse gas cell distribution in baked breads. Vertically ellipsoidal pores were observed in control samples, while round or large, long pores were observed in yukone bread made with gelatinised starches. The addition of gelatinised starches has been shown to significantly increase the ratio of pore area to total area, the average pore area, and the average pore circularity [33]. A decrease in the uniformity of the breadcrumb pores has also been observed, but this was not reflected by graphical analysis. Cakes prepared with gelatinised banana starches have a less porous structure with fewer air cells [39]. The pictures of GFRB crumbs are shown in Figure 2a,b. Convention, identification, and analysis of the image of GFRB pore distribution is shown in Figure 2c, and relative results are listed in Table 5. A decrease in pore size homogeneity can be observed by the naked eyes from Figure 2a,b. However, this decrease in the homogeneity was not reflected by the image analysis methods used in this study. A small, but non-significant, decrease (*p* > 0.05) in the number of pores was observed in breadcrumbs made with PGRF in this study (Table 5). Notably, significant increases in the average pore area, total pore area, and ratio of pore area to total area were observed with the addition of PGRF (*p* < 0.05). The higher average pore area and total pore area implied that the gas-holding capacity of the bread batter was improved by adding PGRF during fermentation and baking, which is consistent with the specific volume results discussed above. These effects on pores may be the result of gelatinised starch granules helping to sustain the gas pressure during fermentation, such that the gas cells were quickly captured by the PGRF during expansion at low temperature during the early stage of baking. A higher ratio of pore area to total area indicates an increase in the ratio of bread volume to the volume of dough materials and a decrease in the average thickness of the crumb grain network, which were not reflected in crumb image analysis but can be verified by decreases in bread hardness and chewiness, as discussed above.

### 3.6. Microstructure of GFRB Batter

**Figure 3:**
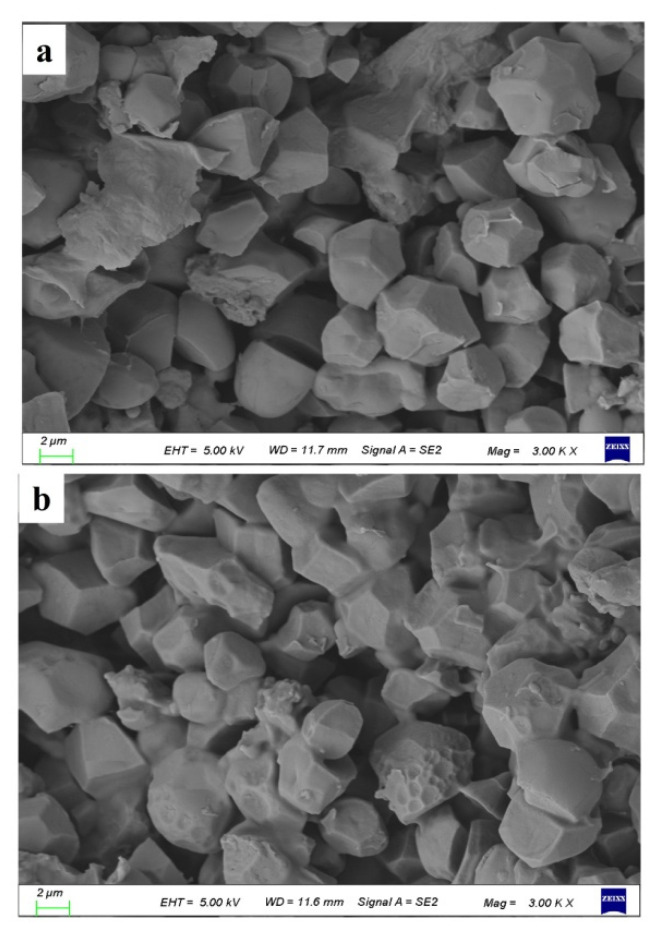
Effect of pre-gelatinisation on the microstructure of gluten-free rice bread batter. (**a**) Control batter; (**b**) batter with pre-gelatinised rice flour.

Pre-gelatinised starches, prepared by thermal, chemical, or mechanical techniques, which are capable of causing the starch granules to swell are generally soluble in cold water [40]. The solubility of pre-gelatinised amylose-rich rice starch under ambient conditions is reported to be close to 80% [41]. Pre-gelatinised starches also provide some application advantages, such as film-forming ability, which is particularly advantageous for applications requiring the formation of moisture and air barriers. In the production of GFRB, pre-gelatinised rice starch helps to trap the CO_2_ produced during fermentation and to resist the pressure caused by CO_2_ expansion in the early stage of baking. As shown in Figure 3a, rice starches were distributed loosely in the control bread batter. Natural starch has polygonal, round granules, and unlike pre-gelatinised starch, it cannot absorb water and increase its viscosity at ambient temperature [42]. Kim et al. [43] reported that upon cooling starch paste, the amylose that leaches out forms a three-dimensional network with the swollen granules embedded in the matrix after gelatinisation. Changes in starch granules cause changes in the rheological behaviour of starch paste and gel, and swollen starch granules form a closely packed gel structure with high shear resistance [43]. Similar closely packed gel structures were also observed in this study (Figure 3b). This closely packed gel structure occasionally forms a constant continual network and can thus be considered an alternative to use in weak flours or those with a low protein content. These starch properties can also control the rheological and textural properties of the final products [44]. Additionally, surface indentation which can be found in annealing treated starch can also observed in pre-gelatinised rice starch, and this may be attributed to the high water/flour ratio used during pre-gelatinisation.

### 3.7. Gelatin Properties of GFRB Batter

**Figure 4:**
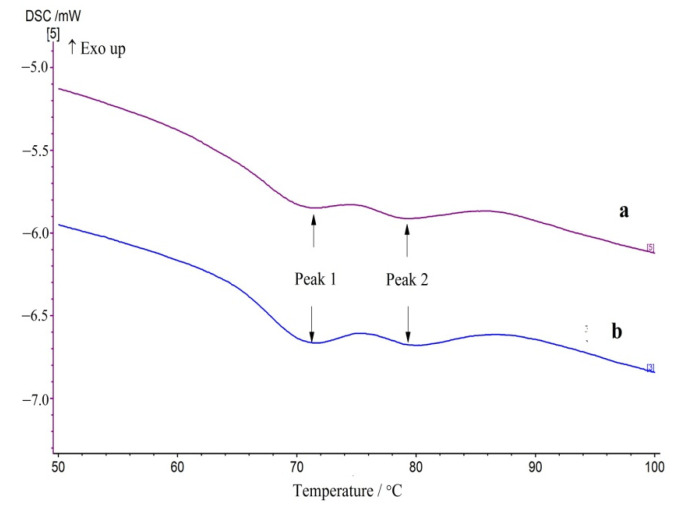
Gelation curve of gluten-free rice bread batter. (**a**) Control batter; (**b**) batter with pre-gelatinised rice flour.

The most important parameters of rice flour when defining breadmaking performance of GFRB are the water binding capacity, swelling power, swelling volume, peak gelatinisation temperature, final gelatinisation temperature, and gelatinisation enthalpy [8]. Thus, the gelation curves of GFRB batters were recorded and results are shown in Figure 4, and the relative gelation parameters are listed in Table 6. A significantly higher onset temperature was observed in peak 1 (*p* < 0.05). No significant differences were observed in the peak temperature, final temperature, or enthalpy value of peak 1, although a slightly higher peak temperature and lower enthalpy were observed (*p* > 0.05). In peak 2, no significant differences were observed in any of the gelation parameters (*p* > 0.05). A shift in the onset pasting temperature has previously been observed in hydrothermally treated rice and corn flours [21]. It was reported that the granules with the least stable crystallites melt first and absorb free water from the ungelatinised granules. Consequently, the water content of the ungelatinised granules decreases, which results in an increase in the melting point. That is, a higher temperature is required to gelatinise the remaining intact starch granules as there is less water available. In addition, the increase in water adsorption and swelling capacity of pre-gelatinised starch in our study led to an increased initial viscosity of the flour and also contributed to the increase in onset temperature. The lack of a significant effect of PGRF on the other gelation parameters may have been caused by the low percentage (1.0%) of PGRF used in this study.

### 3.8. TGA-SDTA of GFRB Batter

Change in the physicochemical, pasting, and rheological properties of starch after pre-gelatinisation were investigated to explain changes in the quality of baked products supplemented with pre-gelatinised flours and starches [45]. The higher water-binding and swelling capacity, the increased initial viscosity, and the decreased viscosity during heating [21] of pre-gelatinised starch are often used to explain the higher specific volume, higher moisture content and slower ageing speed of baked products, respectively, especially leavened ones. Gelatinised flour is thought to act as a hydrocolloid in dough because of a weakened association between starch components during pre-gelatinisation, with boiling resulting in a high water-binding capacity. A higher water-binding capacity has been reported for pre-gelatinised gayam (*Inocarfus fagifer* Forst.) flour compared to the natural flour [18], due to a greater proportion of amorphous chains available to interact with water. Dough viscosity increases through the hydroxyl groups of the hydrocolloidal structure, enabling more interactions with water molecules by hydrogen bonding [46].

**Figure 5:**
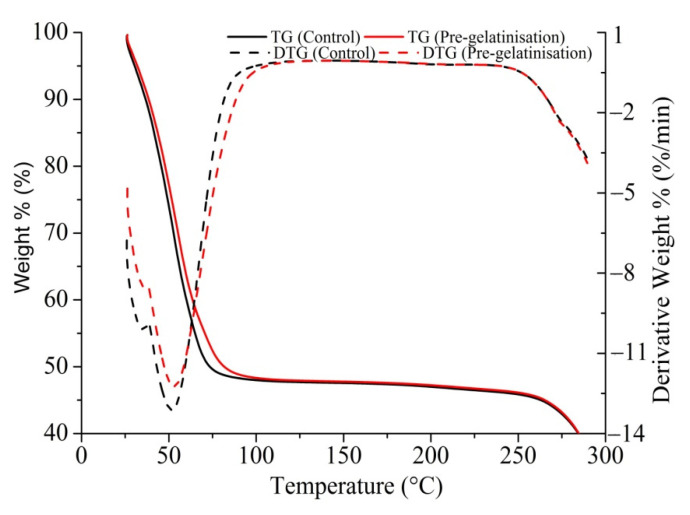
Thermogravimetric analysis/simultaneous differential thermal analysis curve of gluten-free rice bread batter.

However, water-binding capacity, swelling capacity, and pasting properties are generally measured at relatively low temperatures (25 °C, 25 °C, and 95 °C, respectively) compared to baking temperature. It is difficult to accurately determine the role of pre-gelatinised flour during baking. Thus, TGA-SDTA was used in this study to mimic the heating process during baking and to monitor changes in batter weight to investigate the effect of PGRF on GFRB. Results are shown in Figure 5. It can be seen from the TGA curves that the weight decreased in a similar pattern throughout the heating process for the pre-gelatinised bread batter and control batter samples, but the rate of decrease was not the same between these samples as the pre-gelatinised bread batter had a greater weight after heating. The rate of weight loss changed at approximately 61.57 °C, as a crossing over was observed in the derivative weight curves. A faster decrease in weight was observed in the pre-gelatinised bread batter than in the control batter before 61.57 °C. When the temperature was higher than 61.57 °C, a slower decrease in weight was observed in the pre-gelatinised bread batter than in the control batter. Two derivative weight peaks were observed at 35.29 °C and 51.72 °C in control bread batter and 36.78 °C and 52.95 °C in pre-gelatinised bread batter, respectively. Weight loss was mainly caused by the evaporation of water and the escape of CO_2_. This implies that the effect of pre-gelatinisation on the water-binding capacity of rice flour during bread making was more complex than its effect at relatively low temperatures. The addition of PGRF caused the water in the bread batter to evaporate more readily in the early stage of baking, but limited evaporation in the following higher-temperature stages.

## 4. Conclusions

Pre-gelatinisation parameters, including pre-gelatinisation temperature, ratio of used water to the total water, the substitution ratio of PGRF, and the pre-gelatinisation time were optimised, and the effect of PGRF on the quality of GFRB and its batter was studied. The results showed that the optimal PGRF was obtained when 50% batter water was mixed with 1.0% rice flour, and the mixture was heated at 80 °C for 2 min. The optimal pre-gelatinisation parameters of rice flour were different from those normally used for water roux starter, rice porridge, and pre-gelatinised wheat flour. Supplementation with PGRF significantly improved the properties of GFRB by affecting bread baking properties, textural properties, colour, and crumb grain features. These effects were mainly caused by the more closely packed gel structure of rice starch in the bread batter, the higher onset temperature during gelatinisation, and the complex effect of PGRF on water-binding capacity in bread batter during the baking process. Together with major incidences of CD, demand for high quality GF foods has increased in recent years, followed by increasing interest in GF alternative grains. However, knowledge of the processing properties of alternative GF grains and their effect on GF baked products, such as pre-gelatinisation parameters, pasting properties, and baking properties, is still limited as properties varied with grain variety. Thus, more research should be carried out on the processing properties before they are used, and emphasis should be placed on new techniques such as flour pre-gelatinisation to obtain GF foods with improved quality.

## Figures and Tables

**Table 1:** Pre-gelatinisation parameters of rice flour.

Parameters	Levels								
	1	2	3	4	5	6	7	8	9
Pre-gelatinisation temperature (°C)	60	70	80	90	100	-	-	-	-
Substitution rate of pre-gelatinised rice flour (PGRF) (%)	0	0.5	1	1.5	2	3	5	7	10
Ratio of water used for Pre-gelatinisation (%)	25	50	75	100	-	-	-	-	-
Pre-gelatinisation time (min)	0	1	2	3	5	10	-	-	-

**Table 2:** Effect of pre-gelatinisation on the baking properties of gluten-free rice bread.

Samples	Loaf Volume (mL)	Specific Volume (mL/g)	Baking Loss (%)	Moisture Content (%)
Control	262.00 ± 5.10 ^a,1^	3.57 ± 0.08 ^a^	26.97 ± 0.47 ^b^	51.95 ± 0.05 ^a^
Pre-gelatinisation	308.33 ± 2.89 ^b^	4.05 ± 0.11 ^b^	25.04 ± 0.87 ^a^	52.25 ± 0.34 ^a^

^1^ Mean value ± SD with different superscript letters (^a^ and ^b^) in the same column are significantly different (*p* < 0.05).

**Table 3:** Effect of pre-gelatinisation on the texture properties of gluten-free rice bread.

Samples	Hardness (N)	Adhesiveness (N.mm)	Springiness (mm)	Chewiness (mJ)
Control	4.89 ± 0.62 ^a,1^	0.017 ± 0.005 ^a^	2.92 ± 0.12 ^a^	9.85 ± 1.24 ^a^
Pre-gelatinisation	4.35 ± 0.58 ^b^	0.017 ± 0.003 ^a^	2.83 ± 0.09 ^b^	8.94 ± 0.97 ^b^

^1^ Mean value ± SD with different superscript letters (^a^ and ^b^) in the same column are significantly different (*p* < 0.05).

**Table 4:** Effect of pre-gelatinisation on the crumb colour of gluten-free rice bread.

Samples	L*	a*	b*
Control	72.70 ± 0.73 ^a,^^1^	8.09 ± 0.56 ^a^	12.54 ± 0.78 ^a^
Pre-gelatinisation	71.94 ± 0.90 ^b^	8.26 ± 1.26 ^a^	8.93 ± 0.51 ^b^

^1^ Mean value ± SD with different superscript letters (^a^ and ^b^) in the same column are significantly different (*p* < 0.05).

**Table 5:** Crumb grain features obtained by image analysis.

	Average Pore Area (mm^2^)	Number	Ratio	Total Pore Area (mm^2^)
Control	464.73 ± 10.73 ^a^	406.20 ± 11.80 ^a^	038 ± 0.01 ^a^	188,705.20 ± 4026.30 ^a^
Pre-gelatinisation	490.11 ± 14.09 ^b,1^	400.40 ± 16.23 ^a^	0.39 ± 0.01 ^b^	196,073.40 ± 3831.07 ^b^

^1^ Mean value ± SD with different superscript letters (^a^ and ^b^) in the same column are significantly different (*p* < 0.05).

**Table 6:** Gelation parameters of gluten-free rice bread batter.

Samples		Onset Temperature (°C)	Peak Temperature (°C)	Final Temperature (°C)	ΔH (J/g)
Peak 1	Control	64.2 ± 0.14 ^a,1^	69.95 ± 0.21 ^a^	74.8 ± 0.14 ^a^	2.52 ± 0.24 ^a^
	Pre-gelatinisation	64.8 ± 0.28 ^b^	70.35 ± 0.21 ^a^	74.45 ± 0.64 ^a^	2.13 ± 0.30 ^a^
Peak 2	Control	75.55 ± 0.07 ^a^	79.3 ± 0.28 ^a^	84.4 ± 0.85 ^a^	0.76 ± 0.02 ^a^
	Pre-gelatinisation	75.85 ± 0.49 ^a^	79.55 ± 0.78 ^a^	83.55 ± 1.34 ^a^	0.64 ± 0.16 ^a^

^1^ Mean value ± SD with different superscript letters (^a^ and ^b^) in the same column of each peak are significantly different (*p* < 0.05).

## Data Availability

The data presented in this study are available on request from the first author. The data are not publicly available due to restrictions (for example, privacy of research participants).

## Supplementary Materials

The following are available online at www.mdpi.com/article/10.3390/foods10112648/s1, Programme S1: MATLAB programme used in the breadcrumb grain feature determination.
