# Supplementary material for: Pre-Gelatinisation of Rice Flour and Its Effect on the Properties of Gluten Free Rice Bread and Its Batter
Source: Foods. 2021 Nov 1;10(11):2648. doi: 10.3390/foods10112648 (PMC8625319; doi:10.3390/foods10112648)
Supplement: Supplementary file 1 [file foods-10-02648-s001.zip › foods-1430889-supplementary.pdf]

```
[I,map]=imread('C:\Users\Administrator\Desktop\name of image');
hold on;subplot(231);imshow (I,map);title('Original image');
I1 =imcomplement (I);% invert
hold on;subplot(232);imshow (I1);title('Complemented image');
I2=rgb2gray(I1);
hold on;subplot(233);imshow(I2);title('Intensity image');
hold on;subplot(234);imhist(I2);title('Resource histogram');
axis([0 120 0 15000]);
level = graythresh(I2);
imgbw = im2bw(I2,level);
hold on;subplot(235);imshow(imgbw);title('Binarization and threshold segmentation');
se=strel ('disk', 1);% Expansion determines the structure of the element
I3=imclose (imgbw,se);% form close
I4=imopen(I3,se);% form open
hold on;subplot(236);imshow(I4);title('Void profile');
I5=I3&I4;
 [r,num]=bwlabel(I5,4);
L=regionprops(r, 'area');
area=cat(1,L. Area);
totalarea=sum(area);
ratio=totalarea/(709*709);
averagearea=sum(area)/num;
```
